# Prior Beliefs Modulate Projection

**DOI:** 10.1162/opmi_a_00042

**Published:** 2021-09-10

**Authors:** Judith Degen, Judith Tonhauser

**Affiliations:** Department of Linguistics, Stanford University; Department of English Linguistics, University of Stuttgart

**Keywords:** experimental semantics, experimental pragmatics, projection

## Abstract

Beliefs about the world affect language processing and interpretation in several empirical domains. In two experiments, we tested whether subjective prior beliefs about the probability of utterance content modulate *projection*, that is, listeners’ inferences about speaker commitment to that content. We find that prior beliefs predict projection at both the group and the participant level: the higher the prior belief in a content, the more speakers are taken to be committed to it. This result motivates the integration of formal analyses of projection with cognitive theories of language understanding.

## INTRODUCTION

Psycholinguistic work has documented several ways in which probabilistic beliefs about the world, often termed *world knowledge*, affect language processing (e.g., Chambers et al., 2002; Hagoort et al., 2004; Hald et al., 2007; Warren & McConnell, 2007), including syntactic ambiguity resolution (e.g., Bicknell & Rohde, 2014; Chambers et al., 2004), reference resolution (e.g., Hanna & Tanenhaus, 2004; Winograd, 1972), genericity (e.g., Tessler & Goodman, 2019), scalar implicature (e.g., Degen et al., 2015), underinformativity implicatures (Kravtchenko & Demberg, 2015), and the production of redundant referring expressions (Degen et al., 2020; Mitchell et al., 2013; Rubio-Fernández, 2016; Sedivy, 2003; Westerbeek et al., 2015). In contrast, formal linguistic research on meaning in the tradition of Montague (1973), which is devoted to specifying how meanings of expressions are computed from the meanings of the parts of the expressions, the way the parts are combined, and the contexts in which the expressions are used, has often sidelined world knowledge as nonlinguistic, encyclopedic knowledge that must enter into the meaning computation, but whose effect has eluded systematic investigation and formalization (for relevant discussion see, e.g., Beaver, 2001; Dowty, 1986; Hobbs, 2019; Peeters, 2000).^1^ In this article, we provide empirical evidence from English that *projection*, a key topic in linguistic research on meaning, is systematically modulated by listeners’^2^ subjective beliefs about the world. This provides further impetus for accounts of meaning computation to include a mechanism for integrating subjective prior beliefs. We provide a sketch of such an account at the end of this article.

To introduce projection, consider first that speakers can present themselves, through their utterances, as believing that a particular content is true, that is, as committed to that content. Listeners, in turn, regularly draw inferences about which content speakers present themselves as committed to. For instance, if a speaker utters *Sam knows that it’s raining*, listeners typically infer that the speaker is committed to the following two contents: (i) the content of the complement (CC) of *know*, that it’s raining; and (ii) the content of the matrix clause, that Sam knows (i). In formal research on meaning, the inference to (ii) is attributed to the speaker having uttered the sentence, and the inference to (i) is attributed to a particular aspect of the lexical meaning of *know*, specifically, that if an individual knows some content *p*, then *p* is true (e.g., Chierchia & McConnell-Ginet, 1990). The puzzle is that the inference to (i) may persist even when the speaker inquires about what Sam knows, as in *Does Sam know that it’s raining?*, or when the speaker denies Sam’s knowledge, as in *Sam doesn’t know that it’s raining*. Because Sam’s knowledge is questioned or even denied in these variants, that is, the inference to (ii) does not persist, these inferences to (i) cannot be attributed to the aforementioned lexical meaning of *know*. This phenomenon of speaker commitment to utterance content that occurs in negated sentences or questions is termed *projection*. Decades of research in formal semantics have aimed to explain why content projects (e.g., Beaver & Geurts, 2014; Langendoen & Savin, 1971).

While content is standardly taken to either project or not (Beaver & Geurts, 2014), recent experimental work suggests that projection is gradient: listeners’ inferences about speaker commitment to utterance content vary in strength. This experimental work suggests that several factors modulate the strength of the inference, including the expression (e.g., *know* vs. *discover* vs. *announce*), the discourse status of the content, and the prosody of the utterance (for an overview see Tonhauser, Beaver, & Degen, 2018). The hypothesis that listeners’ prior beliefs modulate projection was initially put forth by Stevens et al. (2017) and Tonhauser et al. (2018), who observed by-item projection variability for different CCs of clause-embedding predicates like *know* and *discover*. They argued that one source of the observed variability may be that more a priori likely content (*Kim flew to New York*) projects more strongly than less a priori likely content (*Kim flew to the moon*) when realized as the CC of a clause-embedding predicate (as in *Did John discover that Kim flew to New York/the moon?*). This idea can straightforwardly be made sense of under recent Bayesian accounts that treat pragmatic utterance interpretation as a matter of combining uncertain prior beliefs about the world with uncertain beliefs about likely speaker production choices via Bayes’ rule (Degen et al., 2015; Goodman & Frank, 2016): a CC that is more likely a priori (*before* observing an utterance) is also more likely a posteriori (*after* observing an utterance).

There is conflicting evidence for the hypothesis that prior beliefs modulate projection. Support for the hypothesis comes from Mahler (2020), who investigated the projection of politically charged CCs of English clause-embedding predicates. For example, the politically charged content in (1) is that Obama improved/damaged the American economy. The prior probability of the content was manipulated by the speaker (Cindy in [1]) speaking at the club meeting of either the College Republicans or Democrats.

(1)  Cindy, at the College Republicans/Democrats club meeting: Ben doesn’t know that …
  - … Obama improved the American economy.
  - … Obama damaged the American economy.     (Mahler, 2020, pp. 784–785)

Higher prior probability content (e.g., a liberal content like [1a] uttered by a Democrat) was more projective than lower prior probability content (e.g., a liberal content uttered by a Republican).

In contrast, Lorson (2018) did not find empirical support for the hypothesis that listeners’ prior beliefs modulate projection in a study of the projection of the prestate content of the English change of state verb *stop*. Prior probability was manipulated through gender stereotypes reported in Boyce, von der Malsburg, Poppels, and Levy (2018). For instance, because men are more likely than women to be plumbers, the prestate content of (2a), that James has worked as a plumber, was hypothesized to be more projective than the prestate content of (2b), that Linda has worked as a plumber.

(2)  a. Did James stop working as a plumber?
    b. Did Linda stop working as a plumber?        (Lorson, 2018, p. 38)

Several differences between Mahler (2020) and Lorson (2018) could be implicated in the differential support for the hypothesis: (a) the projective content investigated (CCs vs. prestate content of *stop*); (b) stimulus type (negated sentences vs. questions); (c) the manipulation of prior beliefs (political party affiliation vs. gender stereotypes); and (d) how explicitly the prior-manipulating information was provided to participants (statement of political party affiliation vs. use of a male or female name to indicate gender). The two experiments reported on in this article provide additional support for the hypothesis that prior beliefs modulate projection. The experiments included 20 clause-embedding predicates (rather than just 7, as in Mahler, 2020) and the prior belief manipulation involved 20 properties of individuals, rather than just political party affiliation (as in Mahler, 2020), or gender (as in Lorson, 2018). Furthermore, we tested the hypothesis both at the level of the individual and of the group: Experiment 1 investigated the effect of prior beliefs on projection by measuring prior probability and projection in a within-participant design. In Experiments 2a and 2b, prior probability and projection were measured in separate groups, as in Mahler (2020) and Lorson (2018).

## EXPERIMENT 1

This experiment tested whether higher prior probability content is more likely to project than lower probability content. Prior probability and projection ratings were collected for the contents of 20 clauses that realized the complements of 20 clause-embedding predicates^3^

### Methods

#### Participants

Three hundred participants with U.S. IP addresses and at least 99% of previous human intelligence tasks (HITs) approved were recruited on Amazon’s Mechanical Turk platform (ages: 18–82, median: 35.5; 119 female, 179 male, 1 other, 1 undeclared). They were paid $1.80 and took 9.1 minutes on average to complete the experiment.

#### Materials and Procedure

The prior probability and projection of the contents of 20 clauses were measured in separate blocks. Each clause (e.g., *Julian dances salsa*) was paired with two facts between participants: The content of the clause was expected to have a higher prior probability in the presence of one fact (e.g., *Julian is Cuban*) than of the other (e.g., *Julian is German*). See the Supplemental Materials for the full set of clauses and facts.

**Figure 1.**
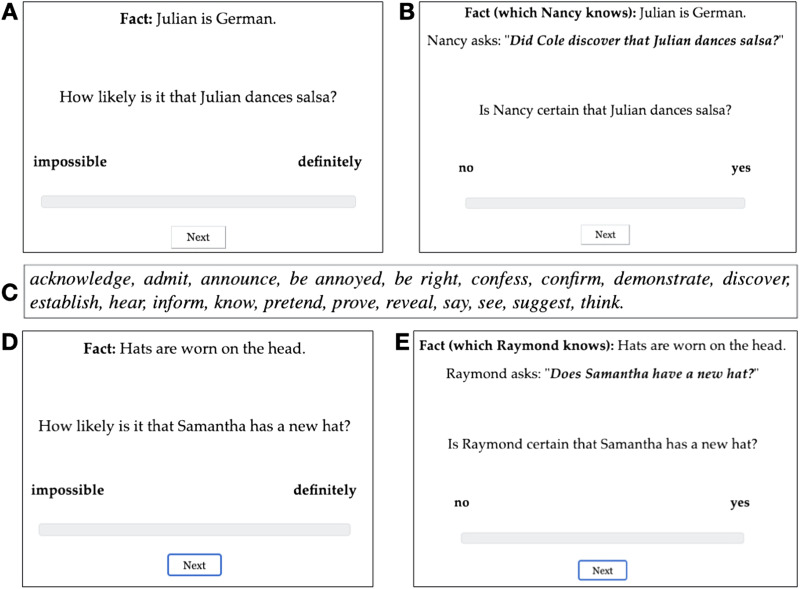
**Example trials and 20 clause-embedding predicates.** **A.** Example target trial in prior block. **B.** Example target trial in projection block. **C.** The 20 clause-embedding predicates. **D.** Example filler trial in prior block. **E.** Example control trial in projection block.

In the prior block, the 20 clauses were realized as the complements of *How likely is it that … ?* questions. As shown in Figure 1A, each target stimulus consisted of one of the two facts for that clause and the *How likely is it that … ?* question. Participants read the fact and assessed the likelihood of the content, given the fact. They gave their responses on a slider marked “impossible” at one end (coded as 0) and “definitely” at the other (coded as 1).

In the projection block, target stimuli consisted of a fact and a polar question that was uttered by a named speaker, as shown in Figure 1B. The polar questions were formed by realizing the 20 clauses as the complements of the 20 clause-embedding predicates in Figure 1C. Participants were told to imagine that they are at a party and that, on walking into the kitchen, they overhear somebody ask somebody else a question. Projection was measured using the “certain that” diagnostic (Djärv & Bacovcin, 2017; Lorson, 2018; Mahler, 2020; Tonhauser et al., 2018): participants were asked to rate whether the speaker was certain of the CC, taking into consideration the fact. They gave their responses on a slider marked “no” at one end (coded as 0) and “yes” at the other (coded as 1). Greater speaker commitment to the CC should result in higher slider ratings.

The projection block also included six control trials, which functioned as attention checks. The content of these stimuli was expected not to project: For example, in Figure 1E, the speaker is not committed to the main clause content, that Samantha has a new hat. The same six main clauses were also used to form 6 filler trials in the prior block; a sample stimulus is given in Figure 1D. These filler stimuli were not used to assess participants’ attention. For the full set of stimuli see the Supplemental Materials.

Each participant’s stimulus set was semi-randomly generated by first randomly pairing up the 20 predicates and clauses. Half of the stimuli were then randomly assigned the respective clause’s higher-probability fact, and half its lower-probability fact. Participants completed a total of 52 trials: 20 target trials in each block, six control trials in the projection block, and six filler trials in the prior block. Each participant completed the same six filler and control trials. Block order and within-block trial order were randomized.

After completing the experiment, participants filled out a short optional demographic survey. To encourage truthful responses, participants were told that they would be paid no matter what answers they gave in the survey.

#### Data Exclusion

Data were excluded based on self-declared non-native speaker status and other criteria given in the Supplemental Materials, leaving 5,720 data points from 286 participants to be analyzed (ages 18–82; median: 35.5; 116 female, 186 male, 1 other, 1 undeclared).

### Results and Discussion

#### Prior Beliefs

**Figure 2.**
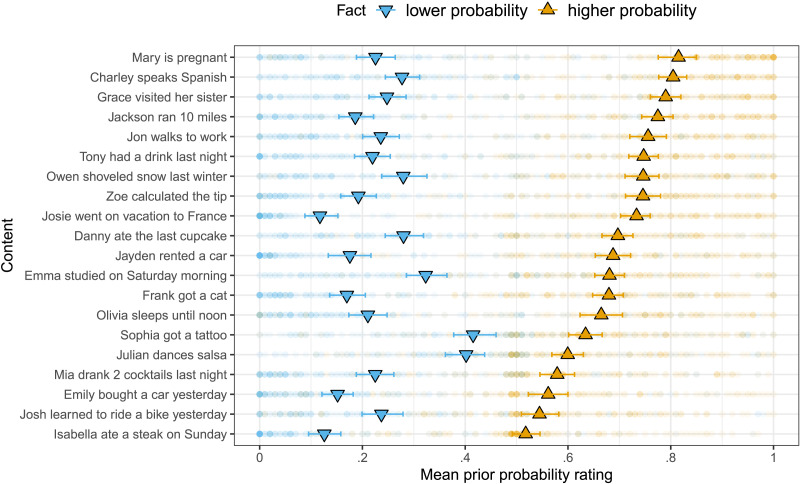
**Mean prior probability by content and fact in Experiment 1.** Error bars indicate 95% bootstrapped confidence intervals. Transparent dots indicate individual participant ratings.

Figure 2 shows the mean prior probabilities of the 20 contents by fact. We conducted a mixed-effects linear regression predicting slider rating from dummy-coded fact type (reference level: “lower probability”) and random by-item and by-participant intercepts and slopes for fact type.^4^ Each content’s mean prior probability was rated as higher when it was presented with its higher probability fact than when it was presented with its lower probability fact (*β* = 0.45, *SE* = 0.01, *t* = 31.12, *p* < .0001). This suggests that the manipulation of the prior probability of the 20 contents was successful.

#### Do Prior Beliefs Modulate Projection?

**Figure 3.**
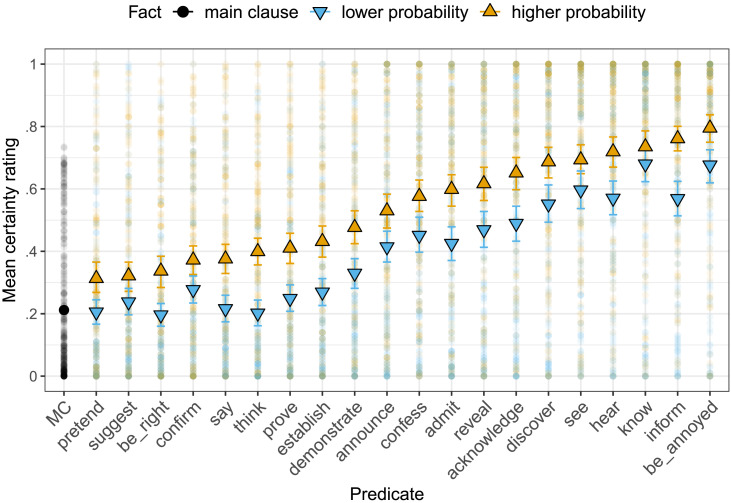
**Mean certainty ratings by predicate and prior probability of the content of the complement in Experiment 1.** Error bars indicate 95% bootstrapped confidence intervals. Light dots indicate participants’ ratings.

Figure 3 shows the mean certainty ratings for the CCs by predicate and by fact, as well as the mean certainty rating for the main clause controls (abbreviated MC). Each predicate/clause combination was rated 5–25 times (mean 14.3). We conducted a mixed effects linear regression predicting certainty ratings from dummy-coded fact type (reference level: “lower probability”) and random by-item and by-participant intercepts and slopes for fact type. The mean certainty ratings were higher for contents presented with higher probability facts than for contents presented with lower probability facts (*β* = 0.14, *SE* = 0.01, *t* = 12.24, *p* < .0001). The same was true when using the group-level by-item mean prior belief as a predictor (*β* = 0.31, *SE* = 0.02, *t* = 12.58, *p* < .0001). This suggests that participants’ prior beliefs about content probability systematically modulated the extent to which they take the speaker to be committed to that content.

We also replicated the by-predicate variability in the projection of the CC observed by Tonhauser and Degen (2020): for instance, the CC of *be annoyed* was more projective than that of *discover*, which in turn was more projective than that of *announce*. The Spearman rank correlation between the mean certainty ratings in Experiment 1 (collapsing over facts) and Experiment 1a of Tonhauser and Degen (2020) is .991; see the Supplemental Materials for a visualization. Experiment 1 thereby also provides further evidence for the systematic influence of the predicate on projection. Crucially, the effect of the prior was observable independently of the predicate.

**Figure 4.**
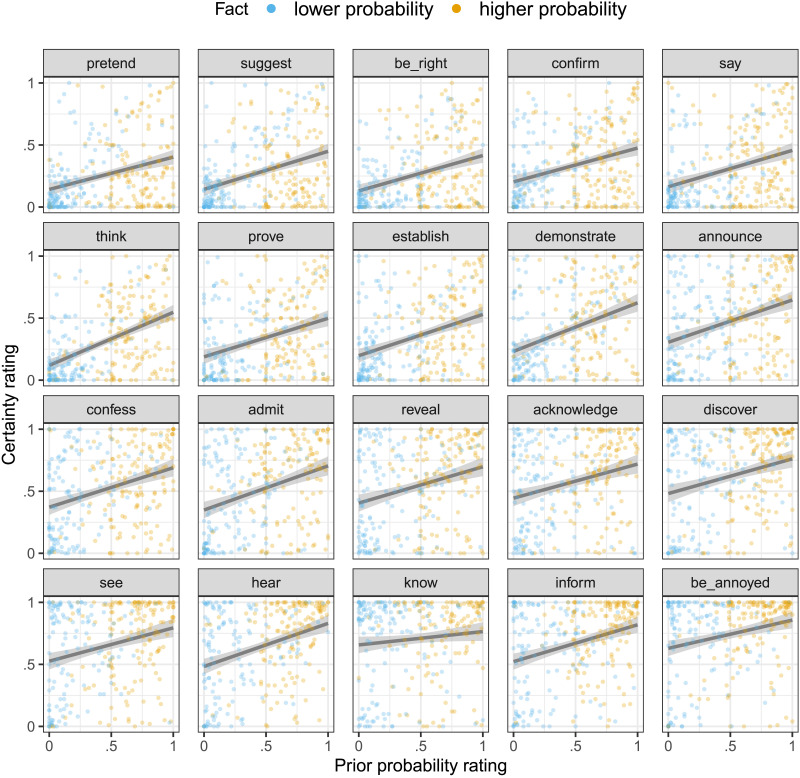
**Certainty ratings against individual prior probability ratings for each predicate in Experiment 1.** Linear smoothers with 95% confidence intervals are overlaid.

Closer inspection of Figure 2 reveals by-participant variability in prior probability ratings, suggesting that individual participants’ prior beliefs may not align with the prior probability classification assumed in Figure 3. For example, given a particular content (*Julian dances salsa*), it is possible that one participant’s prior probability rating was lower than that of another participant, even though the first participant was presented with the higher probability fact (*Julian is Cuban*) and the second one with the lower probability fact (*Julian is German*). Figure 4 shows participants’ certainty ratings by their individual prior probability ratings. To investigate whether prior beliefs modulate projection at the by-participant level, we conducted the same mixed-effects analysis reported above, but used participants’ individual, continuous prior probability ratings as the fixed effect prior predictor. Again, higher prior–probability CCs were more likely to project (*β* = 0.28, *SE* = 0.02, *t* = 13.85, *p* < .0001). This suggests that prior beliefs modulate projection even at the by-participant level. A Bayesian information criterion (BIC) model comparison revealed that the individual-level model better captured the variance in the data (categorical model BIC: 2654; group-level model BIC: 2586; individual-level model BIC: 2291),^5^ suggesting that individual listeners’ prior beliefs systematically modulate the extent to which they take the speaker to be committed to a content: the more they believe it, the more they take the speaker to believe it.

The results of Experiment 1 provide empirical support for the hypothesis that higher prior probability content is more likely to project. It is possible, however, that the within-participant design resulted in participants’ responses on either block influencing their responses on the other block. To guard against this possibility, we replicated Experiment 1 by collecting prior probability and projection ratings from different groups.

## EXPERIMENT 2

Experiments 2a and 2b measured the prior probability and the projection of the 20 contents of Experiment 1, respectively.

### Methods

#### Participants

Participants with U.S. IP addresses and at least 99% of previous HITs approved were recruited on Amazon’s Mechanical Turk platform. The 95 participants in Experiment 2a (ages: 21–75, median: 33; 45 female, 50 male) were paid 55 cents and took 3.9 minutes on average to complete the experiment. The 300 participants in Experiment 2b (ages: 21–72, median: 36; 145 female, 154 male, 1 undeclared) were paid 85 cents and took 7.1 minutes on average.

#### Materials and Procedures

The target stimuli of Experiment 2a were identical to those of the prior block of Experiment 1. Each participant saw two control stimuli as attention checks (see the Supplemental Materials). The materials of Experiment 2b were identical to those of the projection block of Experiment 1. Trial order in both experiments was random. The procedures of Experiments 2a and 2b were identical to those of the prior and projection blocks of Experiment 1, respectively.

#### Data Exclusion

We excluded data based on the criteria given in the Supplemental Materials, leaving data from 75 participants to be analyzed in Experiment 2a (1,500 data points; ages 21–75; median: 35; 34 female, 41 male) and from 266 participants in Experiment 2b (5,320 data points; ages 21–72; median: 36; 129 female, 136 male, 1 undeclared).

### Results and Discussion

#### Prior Beliefs

**Figure 5.**
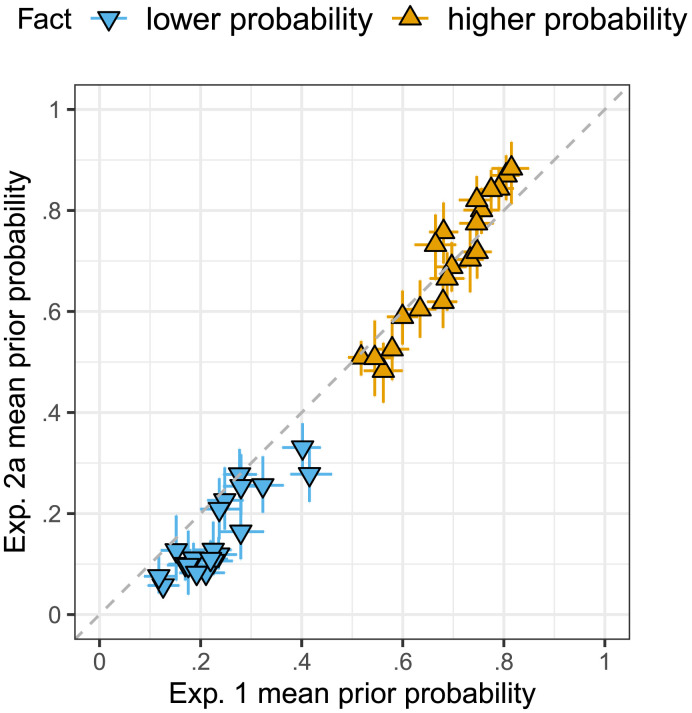
**Mean prior probability ratings in Experiment 2a against those of Experiment 1.** Error bars indicate 95% bootstrapped confidence intervals.

Experiment 2a successfully replicated the prior probability manipulation of Experiment 1: contents were rated as more likely when presented with a higher probability fact (*β* = 0.54, *SE* = 0.04, *t* = 15.07, *p* < .0001). Figure 5 shows contents’ mean prior probability ratings in Experiment 2a against those of Experiment 1. The Spearman rank correlation was very high, at *r* = .977. For a visualization of the by-content prior ratings see the Supplemental Materials.

#### Do Prior Beliefs Modulate Projection?

**Figure 6.**
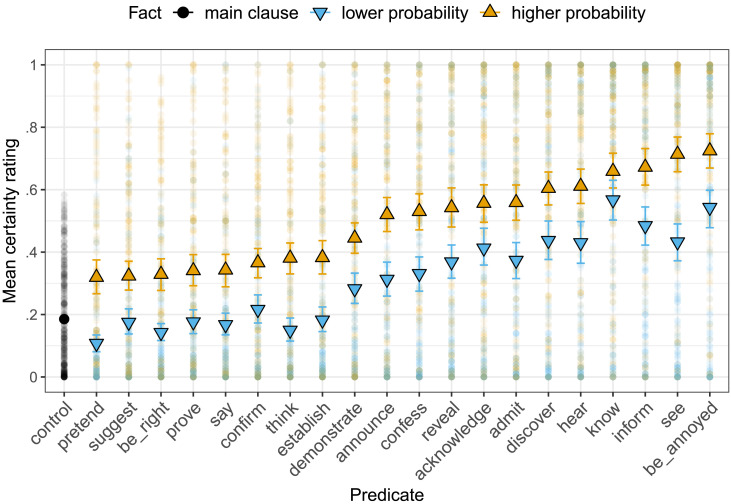
**Mean certainty ratings by predicate and prior probability of the content of the complement in Experiment 2b.** Error bars indicate 95% bootstrapped confidence intervals. Light dots indicate participants’ ratings.

Each predicate/clause combination was rated 4–27 times (mean 13.3). Mean certainty ratings were higher for contents presented with higher prior probability facts than for contents presented with lower prior probability facts (see Figure 6). This was true when the prior predictor was entered as a categorical predictor (reference level: “lower probability”; *β* = 0.18, *SE* = 0.01, *t* = 12.81, *p* < .0001) and when it was entered as a continuous predictor representing group-level prior means (*β* = 0.34, *SE* = 0.03, *t* = 13.27, *p* < .0001). Thus, Experiment 2b replicates the critical result of Experiment 1 that prior content probability modulates its projection.^6^ The replication suggests that the result of Experiment 1 is not an artifact of the within-participant design of Experiment 1.

## GENERAL DISCUSSION AND CONCLUDING REMARKS

We tested whether listeners’ prior beliefs modulate projection. While previous research on this question has yielded conflicting results (Lorson, 2018; Mahler, 2020), we showed in two experiments that content is more likely to project the more a priori likely it is, thus confirming the results of Mahler (2020) and expanding on them in several ways. First, while Mahler (2020) manipulated only the political party affiliation of the speaker, the manipulation in Experiments 1 and 2 relied on 20 distinct properties of individuals (e.g., whether Julian is more likely to dance salsa if he is German or Cuban). Thus, the results of Experiments 1 and 2 suggest a general effect of prior beliefs on projection. Second, our experiments show that prior beliefs modulate projection for a wider cross-section of clause-embedding predicates, including cognitive (e.g., *know*), emotive (e.g., *be annoyed*), communication (e.g., *announce*), and inferential (e.g., *prove*) predicates. Thus, projection joins the many linguistic phenomena that are systematically modulated by prior beliefs (Bicknell & Rohde, 2014; Chambers et al., 2004; Degen et al., 2015; Hagoort et al., 2004; Hald et al., 2007; Kravtchenko & Demberg, 2015; Sedivy, 2003; Tessler & Goodman, 2019; Warren & McConnell, 2007; Westerbeek et al., 2015). Finally, the within-participant design of Experiment 1 shows that individuals’ gradient prior beliefs better predict projection than gradient group-level beliefs,which in tum better predict projection than binary, categorical group-level beliefs (as investigated by Mahler, 2020). This suggests that at least some by-participant variability observed in previous projection experiments (see, e.g., Tonhauser et al., 2018; Tonhauser & Degen, 2020) may be due to participants assigning different prior probabilities to investigated content.

Does our work help explain the discrepancy in findings between the work of Mahler (2020) and Lorson (2018)? In the introduction, we raised four possibilities for the observed differences: (a) the projective content investigated (CCs vs. prestate content of *stop*); (b) stimulus type (negated sentences vs. questions); (c) the manipulation of prior beliefs (political party affiliation vs. gender stereotypes); and (d) how explicitly the prior-manipulating information was provided to participants (statement of political party affiliation vs. use of a male or female name to indicate gender). Our work used (a) CCs; (b) questions; (c) introduced novel properties of individuals; and (d) introduced prior-manipulating information via explicit statement of facts. Thus, our work rules out option (b)—that prior beliefs don’t affect projection out of questions—but not the other possibilities, which should be investigated in future work.

Our results have two broader implications. First, they suggest that the purview of projection analyses is wider than assumed by current analyses, which typically limit their attention to a narrow subset of clause-embedding predicates, like factive ones (e.g., Abrusán, 2011, 2016; Heim, 1983; Romoli, 2015; Simons et al., 2017; van der Sandt, 1992). Rather than being categorical and limited to factive predicates, projection inferences are systematically drawn to varying degrees as a function of at least predicate identity and prior beliefs about content. Second, the results motivate the development of projection analyses that consider listeners’ variable subjective beliefs about the world. Given the gradient nature of the measured (prior and posterior) beliefs and the uncertainty inherent in the different factors that have been shown to modulate projection (e.g., at-issueness, prosody), probability theory suggests itself as a representational framework within which to model projection. To date, only few probabilistic models of projection have been developed (Qing, Goodman, & Lassiter, 2016; Stevens et al., 2017). In these models, projection is the result of listeners’ reasoning about the common ground that the speaker is assuming and the likely question that was being addressed, respectively. While neither investigated the effect of prior beliefs explicitly, both models are couched within the Rational Speech Act (RSA) framework (Franke & Jäger, 2016; Goodman & Frank, 2016), which standardly assumes that utterance interpretation is modulated by listeners’ prior beliefs. The RSA framework is thus equipped to capture the effects reported here. We see the implementation of projection analyses within RSA as a promising avenue for formalizing the intricate interplay of semantic and pragmatic factors in the projection of contents of complements of clause-embedding predicates, including the conventional contribution of predicates, content at-issueness, and subjective prior beliefs about content.

## ACKNOWLEDGMENTS

For helpful comments on the research presented here, we thank the audiences at the 2018 Annual Meeting of XPRAG.de and at the University of Tübingen.

## FUNDING INFORMATION

JT, National Science Foundation (https://dx.doi.org/10.13039/100000001), Award ID: BCS-1452674.

## AUTHOR CONTRIBUTIONS

JD: Conceptualization: Equal; Formal analysis: Lead; Methodology: Equal; Visualization: Lead; Writing - Original Draft: Equal; Writing - Review & Editing: Equal. JT: Conceptualization: Equal; Formal analysis: Supporting; Methodology: Equal; Visualization: Supporting; Writing - Original Draft: Equal; Writing - Review & Editing: Equal.

## Notes

^1^ Because *knowledge* implies justified true belief but subjective beliefs need not be accurate to affect language processing in systematic ways, we henceforth avoid the term *world knowledge* and instead refer to *(subjective prior) beliefs about the world*.^2^ We include readers, writers, and signers in the terms *listener* and *speaker*.^3^ The experiments, data and R code for generating the figures and analyses of the experiments reported on in this article are available at https://github.com/judith-tonhauser/projective-probability. Experiment 1 was preregistered: https://osf.io/vd9ru/. All experiments were conducted with approval from the institutional review board of The Ohio State University and informed consent was obtained.^4^ All analyses were conducted in R (R Core Team, 2016) using the lme4 package (Bates et al., 2015).^5^ The BIC model comparisons were not preregistered, so we also ran Akaike information criterion (AIC) model comparisons as a robustness check. The results were qualitatively identical (categorical model AIC: 2607; group-level model AIC: 2539; individual-level model AIC: 2244).^6^ 28 participants took Experiment 2b after taking Experiment 2a two weeks before. Analyses that excluded these participants’ data did not change the results. Experiment 2b also replicated the result of Tonhauser and Degen (2020) that there is by-predicate variability in the projection of the CC; see the Supplemental Materials.
