# Supplementary material for: Prior Beliefs Modulate Projection
Source: Open Mind (Camb). 2021 Sep 10;5:59–70. doi: 10.1162/opmi_a_00042 (PMC8563063; doi:10.1162/opmi_a_00042)
Supplement: Supplementary file 1 [file opmi-05-59-s001.pdf]

Prior beliefs modulate projection – Supplementary Materials

Judith Degen[•] and Judith Tonhauser[◦]

[•]Stanford University
[◦]University of Stuttgart

June 12, 2021

A Experiment 1: Target and control stimuli

This list shows the 20 clauses of the target stimuli alongside their lower and higher probability facts, respectively:

1. Mary is pregnant. Facts: Mary is a middle school student / Mary is taking a prenatal yoga class
2. Josie went on vacation to France. Facts: Josie doesn't have a passport / Josie loves France
3. Emma studied on Saturday morning. Facts: Emma is in first grade / Emma is in law school
4. Olivia sleeps until noon. Facts: Olivia has two small children / Olivia works the third shift
5. Sophia got a tattoo. Facts: Sophia is a high end fashion model / Sophia is a hipster
6. Mia drank 2 cocktails last night. Facts: Mia is a nun / Mia is a college student
7. Isabella ate a steak on Sunday. Facts: Isabella is a vegetarian / Isabella is from Argentina
8. Emily bought a car yesterday. Facts: Emily never has any money / Emily has been saving for a year
9. Grace visited her sister. Facts: Grace hates her sister / Grace loves her sister
10. Zoe calculated the tip. Facts: Zoe is 5 years old / Zoe is a math major
11. Danny ate the last cupcake. Facts: Danny is a diabetic / Danny loves cake
12. Frank got a cat. Facts: Frank is allergic to cats / Frank has always wanted a pet
13. Jackson ran 10 miles. Facts: Jackson is obese / Jackson is training for a marathon
14. Jayden rented a car. Facts: Jayden doesn't have a driver's license / Jayden's car is in the shop
15. Tony had a drink last night. Facts: Tony has been sober for 20 years / Tony really likes to party with his friends
16. Josh learned to ride a bike yesterday. Facts: Josh is a 75-year old man / Josh is a 5-year old boy
17. Owen shoveled snow last winter. Facts: Owen lives in New Orleans / Owen lives in Chicago
18. Julian dances salsa. Facts: Julian is German / Julian is Cuban
19. Jon walks to work. Facts: Jon lives 10 miles away from work / Jon lives 2 blocks away from work
20. Charley speaks Spanish. Facts: Charley lives in Korea / Charley lives in Mexico

In the target stimuli of the projection block of Exp. 1, eventive predicates, like *discover* and *hear*, were realized in the past tense and stative predicates, like *know* and *be annoyed*, were realized in the present tense. The direct object of *inform* was realized by the proper name *Sam*. Each clause-embedding predicate

was paired with a unique subject proper name. The speaker of the target stimuli was realized by a randomly sampled unique proper name.

The following list shows the six clauses that were used in the control and filler stimuli of Exp. 1, with their facts. In the prior block, these six clauses were embedded under *How likely is it that… ?*. The projection block featured polar questions variants of the clauses.

1. Zack is coming to the meeting tomorrow. Fact: Zack is a member of the golf club.
2. Mary's aunt is sick. Fact: Mary visited her aunt on Sunday.
3. Todd played football in high school. Fact: Todd goes to the gym 3 times a week.
4. Vanessa is good at math. Fact: Vanessa won a prize at school.
5. Madison had a baby. Fact: Trish sent Madison a card.
6. Hendrick's car was expensive. Fact: Hendrick just bought a car.

B Data exclusion

Table A1 presents how many participants' data were excluded from the analyses based on the exclusion criteria. The first column records the experiment, the second ('recruited') how many participants were recruited, and the final column ('remaining') how many participants' data entered the analysis. The 'Exclusion criteria' columns show how many participants' data were excluded based on the two exclusion criteria:

- 'language': Participants' data were excluded if they did not self-identify as native speakers of American English.
- 'controls': In Exps. 1 and 2b, participants' data were excluded if their response mean on the 6 control items was more than 2 sd above the group mean. In Exp. 2a, participants' data were excluded if their response to (1a) was more than 2 sd below the group mean or if their response to (1b) was more than 2 sd above the group mean.

		Exclusion criteria		
	recruited	language	controls	remaining
Exp. 1	300	3	11	286
Exp. 2a	95	8	12	75
Exp. 2b	300	23	11	266

Table A1: Data exclusion in Exps. 1 and 2

C Projection comparisons

Fig. A1 compares the mean certainty ratings of the predicates and main clause controls in Exp. 1, Exp. 2b, and Tonhauser & Degen, under review Exp. 1a (abbreviated ‘Exp. 1a TD’). The Spearman rank correlations were .986 (Exp. 2b vs. Exp. 1a TD), .988 (Exp. 1 vs. Exp. 2b) and .991 (Exp. 1a TD vs. Exp. 1).

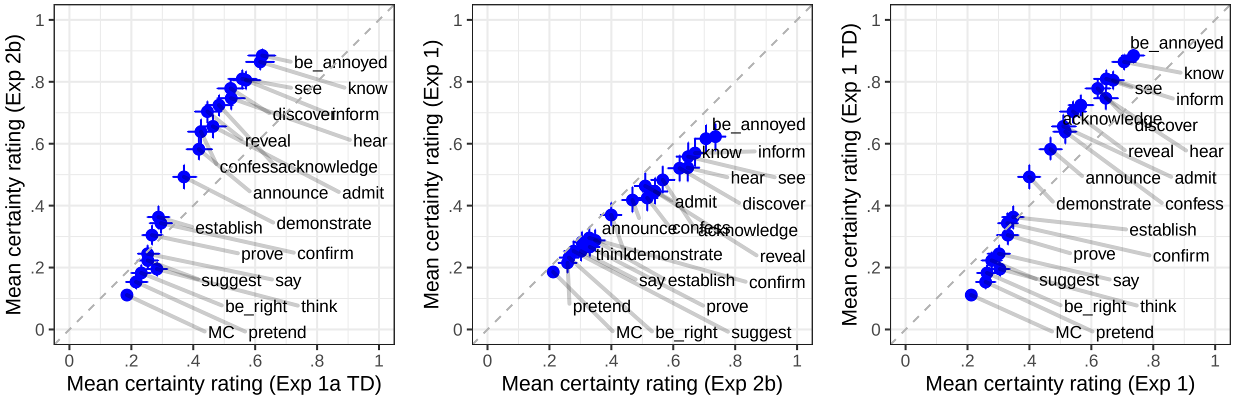

(a) Exp. 2b vs. Exp. 1a TD. (b) Exp. 1 vs. Exp. 2b. (c) Exp. 1a TD vs. Exp. 1.

Figure A1: Comparisons of mean by-predicate certainty ratings from Exp. 1, Exp. 2b, and Tonhauser & Degen’s Exp. 1a (abbreviated ‘Exp. 1a TD’). Error bars indicate 95% bootstrapped confidence intervals. Range of means from current experiments appears compressed compared to that of Tonhauser & Degen because means are collapsed across fact type.

D Experiment 2 supplements

The control items in Exp. 2a are given in (1).

(1) a. **Fact:** Barry lives in Germany.
How likely is it that Barry lives in Europe?

b. **Fact:** Tammy is a rabbit.
How likely is it that Tammy speaks Italian and Greek?

Fig. A2 shows the mean prior probabilities of the 20 contents by fact. Participants' ratings are given as light dots. The mean prior probability rating for each content was higher when the content was presented with the higher probability fact than when it was presented with the lower probability fact.

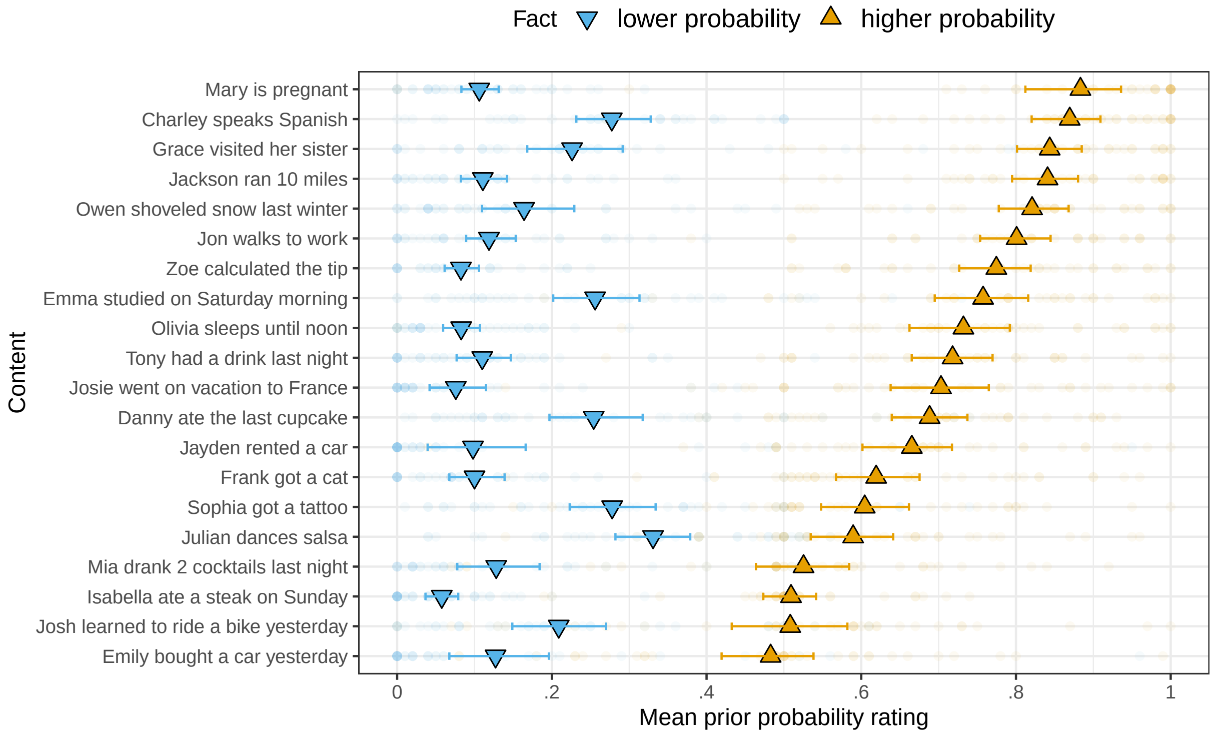

Figure A2: Mean prior probability by content and fact in Exp. 2a. Error bars indicate 95% bootstrapped confidence intervals. Light dots indicate participants' ratings.

References

Tonhauser, J., & Degen, J. (under review). *Are there factive predicates? An empirical investigation.* (Manuscript under review, Stuttgart University and Stanford University, retrieved from `https://ling.auf.net/lingbuzz/005360`)
